# Editorial: Clinical uses and alternative approaches of frailty determination

**DOI:** 10.3389/fphys.2025.1575742

**Published:** 2025-04-11

**Authors:** Kenneth L. Seldeen, John A. Batsis

**Affiliations:** ^1^ Division of Geriatrics, University of Kansas Medical Center, Kansas City, KS, United States; ^2^ Research Service, Veterans Affairs Kansas City Healthcare System, Kansas City, MO, United States; ^3^ Division of Geriatric Medicine, School of Medicine, and Department of Nutrition, Gillings School of Global Public Health, University of North Carolina at Chapel Hill, Chapel Hill, NC, United States

**Keywords:** frailty, aging, physical performance, resilience, functional capacity

Frailty represents a greater vulnerability to stressors that increases an individual’s susceptibility to adverse health outcomes, such as disability, loss of independence, and death. As one ages, the prevalence of frailty increases and affects up to 50% of those aged 85 and older (Clegg et al., 2013). Efforts to characterize and quantify states of frailty took a substantial leap forward with the emergence of frailty assessment frameworks based on physical frailty and deficit accumulation in the early 2000s (Fried et al., 2001; Rockwood and Mitnitski, 2007; Searle et al., 2008). Since then, frailty tools have been correlated with important health outcomes relevant to aging, have been used to evaluate therapeutic benefit, and are now being explored to help scientists understand the underlying biology of frailty (Fried et al., 2001; Rockwood and Mitnitski, 2007; Brivio et al., 2019; Kwak et al., 2020; Ota and Kodama, 2022). Importantly, frailty tools have also found utility in predicting outcomes of medical and surgical interventions and continue to be refined to improve prognosis in older individuals (Ko, 2019; Nidadavolu et al., 2020; Rabelo et al., 2023).

The goal of this Research Topic is to identify alternative tools for the study of frailty. Tools for the rapid assessment of frailty have been reported, including the FRAIL scale (Morley et al., 2012), which can typically be completed in 2–3 min via a five-question survey. Alternatively, a single measure of grip strength or a test of gait speed have also been strongly correlated with frailty and may represent an alternative (Suzuki et al., 2023; Vaishya et al., 2024). However, in addition to clinical workflow issues, as frailty is a multi-factorial syndrome (Heuberger, 2011; Lang et al., 2009; Sezgin et al., 2020), the possibility exists that different tools may capture different aspects of frailty. This point is highlighted by a comparison of frailty tools in mice, which identified differences between physical frailty and deficit accumulation assessment frameworks (Seldeen et al., 2019). The contributions in this Research Topic highlights unique frailty tools along with relationships with important physiological parameters.

The first article by Seldeen et al., identified for the first time correlations between VO_2_max, the 6-minute walk test, and arm strength (using a handheld dynamometer) and frailty in older Veterans. Of interest in this article was that a correlation was observed between VO_2_max and 6-minute walk, but not arm strength–suggesting that the physical performance measures may capture different aspects of frailty (i.e., contribution of strength versus endurance). The next two articles presented different strategies for the use of the clinical frailty scale (CFS, (Rockwood et al., 2005)), a rapid frailty determination tool that scores patients on a nine-point scale based on functional capacity, comorbidity status, and activity of daily living dependency (Rockwood et al., 2005; Pulok et al., 2020). The first, by Zacchetti et al., examined and found that the CFS predicts outcomes in older adults with moderate to severe traumatic brain injuries. In the study, these authors found that patients identified as vulnerable (CFS ≥4) had a staggering 87% mortality at 6 months (versus 30% for non-vulnerable) – demonstrating the utility for risk stratification. The second by Garcia-Chanes et al., employed an adaptation of the CFS designed to allow generation of a CFS score without the need for clinician input. Building on data from the Study on Global Aging and Health (SAGE, (Kowal et al., 2012)), the authors incorporated responses to a wide variety of questions such as activities of daily living, health status, day-to-day activities, self-reported data, etc., which then used a classification tree to generate a score on a seven-point scale. Using this tool the authors identified an intricate relationship between frailty and cognitive performance. Both articles demonstrate alternative applications of an existing frailty framework, allowing for new utility in risk stratification and applicability to different data sources.

The fourth article in this Research Topic, by Liu et al., incorporated a simplified five-item frailty score, generated from the presence of comorbidities or need for assistance with activities of daily living, into a nomogram that can be used to predict 1-, 3-, and 5-year survival following radical nephroureterectomy. The validity of the model provided proof of concept for the incorporation of frailty into outcome prediction for medical interventions. The final article in this issue, by Eisenkraft et al., examined a new detection and warning tool to provide timely alerts of real-time deterioration. The device used was a wireless, wearable chest patch monitor that measured heart rate, blood oxygen saturation, respiratory rate, blood pressure, body temperature, and several cardiac parameters every 5 min. In the article the authors described how the new tool increased sensitivity over the current tool, with detection of impending health events nearly 9 h earlier. The concepts described here involving wearable technologies could be applied to frailty detection, given that an estimated 20% of the US population uses fitness trackers (Anderson, 2020).

The wide range of frailty characterization strategies presented in this Research Topic reflects the multi-factorial nature of frailty. Similarly, such tools may also be useful in characterizing resilience, the ability to respond to and recover from physical and cognitive stresses that challenge homeostasis and the “characteristic which determines one’s ability to resist or recover from functional decline following health stressors (Hadley et al., 2017; Whitson et al., 2016)” (e.g., falls, hip fracture, surgery, hospitalization, etc.). Poor resilience is likely to precede frailty and thus must be maintained for optimal functional capacity, healthspan, and quality of life (Varadhan et al., 2018; Finucane et al., 2017; Kuchel, 2018; O'Connell et al., 2018; Brown et al., 2023; Seong et al., 2022; Whitson et al., 2018; Zhang et al., 2023). The development of frailty tools could therefore be further re-purposed to explore their utility in characterizing resilience, thus allowing the detection of susceptibility before the onset of frailty and therefore allowing a greater opportunity for successful intervention.

Taken together, the studies presented in this Research Topic underscore the dynamic and evolving nature of frailty assessment tools. From traditional clinical tools such as the CFS to novel machine learning approaches and real-time physiological monitoring, these advances highlight the expanding utility of frailty measures in predicting health outcomes and guiding medical interventions. As frailty remains a significant determinant of vulnerability in aging populations, continued innovation in assessment strategies will be critical to improving patient care, risk stratification, and therapeutic decision-making. In the future, integrating multimodal frailty assessment tools—such as physical performance measures, self-reported scales, and other emerging technologies such as wearable sensors—may offer a more comprehensive approach to capturing the complexity of frailty. However, there are a number of unanswered questions: 1) what assessment framework should be used and when; 2) what types of methods should be considered; 3) how can these methods be seamlessly integrated into clinical workflows; 4) how can the data obtained from these measures be used without further burdening already burdened clinicians; and 5) what types of interventions should be considered with specific types of data outputs. This is the tip of the iceberg in terms of integration and translation from science to clinical practice. Ultimately, these advances hold the promise of refining early detection, tailoring interventions, and enhancing the quality of life for older adults.
